# Expression of Properdin, the positive regulator of the Complement Alternative Pathway, at the fetal-maternal interface in Preeclampsia

**DOI:** 10.3389/fimmu.2025.1739327

**Published:** 2026-02-04

**Authors:** Hadida Yasmin, Tamali Roy, Chiara Agostinis, Aishwarya Rao, Priyanka Sharma, Miriam Toffoli, Andrea Balduit, Silvia Pegoraro, Samik Bindu, Rajib Prasad, Giuseppe Ricci, Manu Vatish, Taruna Madan, Roberta Bulla, Uday Kishore

**Affiliations:** 1 Department of Zoology, Cooch Behar Panchanan Barma University, Cooch Behar, India; 2 Institute for Maternal and Child Health, IRCCS Burlo Garofolo, Trieste, Italy; 3 Department of Innate Immunity, ICMR-National Institute for Research in Reproductive and Child Health (NIRRCH), Mumbai, India; 4 Hospital Administration & Medical Services Department, Cooch Behar Maharaja Jitendra Narayan Medical College & Hospital, Cooch Behar, India; 5 Department of Medical, Surgical and Health Science, University of Trieste, Trieste, Italy; 6 Nuffield Department of Obstetrics and Gynaecology, University of Oxford, Oxford, United Kingdom; 7 Development Research, Indian Council of Medical Research (ICMR), New Delhi, India; 8 Department of Life Sciences, University of Trieste, Trieste, Italy; 9 Department of Veterinary Medicine, United Arab Emirates University, Al Ain, United Arab Emirates

**Keywords:** complement, alternative pathway, pregnancy, preeclampsia, properdin, inflammation

## Abstract

**Introduction:**

Aberrant complement activation can cause damage to newly formed fetal-derived structures and excessive inflammatory response at the feto-maternal interface, contributing to pregnancy-related complications, including preeclampsia (PE), which is one of the most severe pathologies in new-borns. Properdin is the only known positive regulator of the complement alternative pathway, as it stabilizes the inherently labile C3bBb complex and amplifies its activity. This study describes the presence of properdin in PE and investigates its role in the pathogenesis.

**Methods:**

We examined the distribution and expression of properdin at both the transcript and protein levels in term placental tissue, serum, placental syncytiotrophoblast microvesicles (STBMs), and circulating placental exosomes from PE women compared to healthy mothers, using RT-qPCR, western blot, immunohistochemistry, transmission electron microscopy (TEM), and immunofluorescence. To link properdin levels with alternative pathway complement factors, we also assessed the expression of C3 and C5.

**Results:**

PE placentae showed significantly higher properdin, C3 and C5 at transcript as well as protein levels compared to healthy placentae. Conversely, properdin levels in serum, STBMs, and circulating placental exosomes were lower in PE compared to healthy pregnancies. Immunohistochemical analysis revealed properdin distribution throughout the PE placentae, with higher concentrations at the syncytial knots containing pyknotic nuclei were observed via TEM, along with elevated levels of cleaved caspase 3.

**Discussion:**

Thus, properdin was significantly upregulated in the PE placentae, along with C3 and C5, and might be associated with the apoptotic nuclei inside syncytial knots. This evidence suggests that properdin may trigger complement-mediated damage to the placental barrier, exacerbating the development of PE placentae.

## 1 Introduction

Preeclampsia (PE) is a life-threatening obstetric syndrome, with its worldwide incidence estimated to be 4.6% (1). Each year, around four million mothers are diagnosed with PE, resulting in the deaths of over 70, 000 women and 500, 000 infants across the globe (1, 2). PE is characterized by a sudden onset of hypertension after 20 weeks of gestation, along with proteinuria and maternal organ dysfunction. The International Society for the Study of Hypertension in Pregnancy (ISSHP) classifies PE as preterm (delivery <37 weeks of gestation), term (delivery ≥ 37 weeks of gestation) and post-partum. The clinical heterogeneity of PE is reflected in distinct pathophysiological origins, supporting the widely accepted view that PE is not a single disease. Traditionally, two main subtypes have been defined, early onset (EO) and late onset (LO) PE, which differ in symptom severity and timing of the disease onset. However, recent evidence have highlighted that this temporal classification fails to capture the distinct underlying mechanism of the disease. A more biologically relevant distinction has been proposed, distinguishing placental-derived PE (Type I), driven by defective spiral artery remodelling, from maternal-type PE (Type II), which arises from an inadequate maternal cardiovascular adaptation to pregnancy (3). Predicting PE is challenging because placental and vascular anomalies arise as early as the first trimester of pregnancy but often remain silent until symptoms appear after the 20^th^ week (4, 5). Currently, there is no treatment for PE; the only option to resolve this disease is the delivery of the baby. However, the treatment with low dose aspirin (LDA) before the 16^th^ gestation week significantly reduces the risk of PE onset (6). Thus, finding a reliable biomarker for an early detection of PE is of great importance.

Multiple and overlapping pathological processes leading to PE involve endothelial cell activation, intravascular inflammation, and syncytiotrophoblast stress (7). In PE, an excessive maternal systemic inflammatory response to pregnancy due to innate immune dysfunction (1) leads to aberrant infiltration of leukocytes at the feto-maternal interface, which subsequently releases excessive amounts of pro-inflammatory cytokines (TNF-α, IL-1β IL-6, IL-8, IL-12, and IL-18) and chemokines (CXCL1, CXCL8, CXCL10, CXCL12, CXCL16, and CX3CL1) (8–11). The imbalance between pro-angiogenic [vascular endothelial growth factor (VEGF) and placental growth factor (PlGF)] and anti-angiogenic factors [soluble fms-like tyrosine kinase 1 (sFLt1) and soluble endoglin (sENG)] is a likely consequence of defective trophoblast invasion of the uterine spiral arteries and of dysfunctional vessel remodeling. A lack of proper blood supply and an excess of anti-angiogenic factors create a harmful, inflammatory environment within the uterus and mother’s circulation (11–13). The overactivation of maternal innate immunity and the persistent hypoxia inside the placenta cause apoptosis of the syncytiotrophoblast and the release of high amounts of syncytiotrophoblast-derived extracellular microvesicles (STBMs). In PE, STBMs as well as placenta-derived circulating exosomes are elevated compared to those in a normal pregnancy (14–19). Release of placental debris containing microvesicles, exosomes, and apoptotic nuclei from the disintegrated syncytiotrophoblast layer into the maternal circulation can activate the complement system leading to endothelial damage and consequent breakdown of the feto-maternal tolerance (20–22).

During pregnancy, the complement components usually increase with placental growth to eliminate apoptotic trophoblast cells, which are vital for placental invasion (23, 24). Bulla et al. have previously demonstrated that endothelial cells of the spiral arteries acquire the ability to synthesize C1q during pregnancy; moreover, decidual endothelial cells (DECs) synthesize and express C1q on their surface (25). In human placenta, C1q is produced by invading extravillous trophoblasts (EVTs) at the maternal-fetal interface (26). The biologically active split complement products, such as C3a, C4a and C5a, increase in maternal plasma of a healthy pregnant women, possibly to protect the mother and the foetus from infectious challenges (27). Several studies in both human and animals have indicated that dysregulation of the complement cascade may contribute to the onset of PE (28–38). Alteration in complement protein levels, specifically an increase in C3a, C4d and C5a, along with a decrease in C1q, have been observed in PE. Elevated levels of C3a in PE pregnancies can suggest involvement of the complement alternative pathway (39). However, Belmonte and colleagues have shown that complement components and activation products are deposited in placental tissue obtained from patients with PE, where they undergo local activation via the complement lectin pathway, initiated by ficolin-3 (40).

Properdin, a soluble glycoprotein of ~53 kDa, is the only known positive regulator of the complement alternative pathway, and can contribute to complement over-activation (41). Properdin can bind directly to microbial surface, as well as to apoptotic or necrotic cells, acting as a pattern recognition molecule (42). This leads to recruitment of C3b, thus, initiating the assembly of the alternative pathway C3 and C5 convertases. In addition, properdin can also act as an opsonin, enhancing target mediated phagocytosis and serving as a potent inflammatory modulator (43, 44). Properdin has been shown to be involved in the pathogenesis of several renal diseases, such as glomerulonephritis, systemic lupus erythematosus (SLE), membranoproliferative glomerulonephritis, C3 glomerulopathy, and diabetic nephropathy (45–47). However, placental expression and contribution of properdin to the overactivation of the complement system at feto-maternal interface in PE remains unclear.

This study aims to investigate the role of properdin in the pathogenesis of PE, by examining the distribution and expression of properdin in term placental tissue, serum, STBMs, and circulating placental exosomes.

## 2 Materials and methods

### 2.1 Human subjects and ethics statements

Multiple cohorts of pregnant women at different stages of pregnancy were included in this study. The cohorts of patients are summarized in **Supplementary Figure 1**, **Supplementary Tables 1**–**5**. For each cohort, PE was defined as the new onset of hypertension (Systolic BP ≥ 140mmHg and diastolic BP ≥ 90mmHg at two occasions at least 4 hours apart following 20th week of gestation or a systolic BP ≥ 160mmHg and/or a diastolic BP ≥ 110mm Hg within short interval) and proteinuria (≥300 mg in a 24 hours urine collection, or a protein/creatinine ratio ≥ 0.3 or, a dipstick reading of ≥ 1+ after 20th week of gestation), or in the absence of proteinuria, new onset of thrombocytopenia (Platelet count < 1, 00, 000/µL), or renal insufficiency or impaired liver function or pulmonary edema, or cerebral, or visual symptoms and no other disease and therapy for it (4). For the healthy control (CTRL) group, the inclusion criteria were: no hypertension, no proteinuria, and no other comorbidity or therapy. The matched controls were selected based on age and number of pregnancies. Exclusion criteria for PE group included: chronic hypertension, renal diseases, collagen vascular diseases, cancer, thrombosis, multifetal gestation, diabetes, autoimmune disease, smokers, vesicular mole, infections (bacterial, fungal, viral), and *in vitro* fertilization. Exclusion criteria for the CTRL group comprised hypertension observed after 3 months postpartum, with any kind of ailment or disease, smokers, infections (bacterial, fungal, viral), multifetal gestation, vesicular mole and *in vitro* fertilization. This study did not include pregnant patients with HELLP (hemolysis, high liver enzymes, and low platelet count) syndrome, nor eclampsia. The cohort of pregnant women were selected from Maharaja Jitendra Narayan Medical College and Hospital (MJNMCH; Cooch Behar, West Bengal, India). Fetal villi samples (1 cm wide pieces) of term end placenta (3^rd^ trimester) were collected from PE as well as healthy mothers (CTRL) within an hour following the delivery. For histological profiling, tissues were formalin fixed. For mRNA studies, tissues were taken in RNA Liv solution (Hi Media) that helps in stabilizing the RNA in the tissue. For western blot (WB), tissues were snap frozen and stored at -80^0^ C for further experimentation.

Circulating exosomes were isolated at the National Institute for Research in Reproductive and Child Health, Mumbai, India, collected from the serum samples of early onset PE mothers (PE developed at <34 weeks of gestation) of Nowrosjee Wadia Maternity Hospital (NWMH), Mumbai, India.

Serum samples of PE patients (obtained at the time of PE diagnosis) and matched healthy pregnant women (CTRL, matched for mother and gestational age) were from Institute for Maternal and Child Health, IRCCS “Burlo Garofolo” (Trieste, Italy). Placental histological specimens were collected from both PE and healthy (CTRL) mothers (36).

STBMs, prepared via dual-placental perfusion of PE and healthy placentae, were obtained from the Nuffield Department of Obstetrics and Gynaecology, John Radcliffe Hospital, University of Oxford (United Kingdom), as previously described (33).

The Institutional Ethical Committee (IEC/CBPBU/ZOO/2020/001, MJNMC/IEC/77/2024, IECICMR-NIRRCH 320/2017 and IECNWMH/AP/2017/014) approved the study as per Indian Council of Medical Research guidelines (ICMR). Informed written consent forms were obtained from each patient included in the study prior to the collection of the samples. All the records identifying the patients were kept confidential.

### 2.2 RNA isolation and real time-quantitative PCR

Total RNA was isolated from placental tissues of PE and CTRL women with RNeasy Mini Kit (Qiagen, #74104) using silica membrane spin columns, according to manufacturer’s protocol. The purified RNA was quantified using NanoDrop^®^ spectrometer (Thermo scientific) at 260 nm. 1 ng of total RNA was used to synthesise first-strand cDNA using the Verso cDNA synthesis kit (Thermo Scientific, #AB-1453/A). Real-time quantitative PCR (RT-qPCR) was performed using Bio-Rad CFX Maestro (CFX ConnectTM Real-Time System) and SsoAdvanced Universal SYBR^®^ Green Supermix (Bio-Rad, #1725270). The reaction involved 40 cycles of denaturation (30 sec at 95 °C) and annealing (30 sec at 52 °C to 60 °C; annealing temperature of the primers). The mRNA expression levels of TNF-α, TGF-β1, VEGF, properdin, C3 and C5 were measured following normalizing with the expression of a housekeeping gene (GAPDH). The primer details are listed in **Supplementary Table 6**.

### 2.3 Isolation of circulating placental exosomes using immunoaffinity method

Briefly, 1 mg of washed dynabeads (Invitrogen; #14311D) were incubated with 30 µg of anti-placental alkaline phosphatase (anti-PLAP) antibody (Invitrogen, # MA1-19354) on an end-to-end rotator at 37°C overnight. Next day, the supernatant was discarded by placing the tubes on a magnetic stand and the antibody coupled beads were washed with HB, LB and SB buffers provided in the kit. Serum-derived exosomes (100 µl) from early onset PE (EOPE, n=4) and CTRL (n=4) groups were incubated with anti-human Fc receptor blocking reagent and anti-PLAP antibody bound beads at 4°C overnight. The bound PLAP^+^ exosomes were eluted using 180 µl of elution buffer (0.1M glycine-HCl, pH 2.8) and neutralized with 1M Tris-buffer, pH 8.0. The flowthrough containing PLAP-exosomes were then used for isolating HLA-G^+^ exosomes after incubation with Anti-HLA-G coated dynabeads at 4°C overnight. Finally, PLAP^+^ and HLA-G^+^ exosomes were pooled to obtain circulating placental exosomes. For the extraction of proteins, 250 μl of placental exosomal suspension from PE cases and healthy controls were lysed in 50 μl RIPA buffer (Sigma-Aldrich; #R0278). Lysates were centrifuged at 16000×g for 20 min at 4°C following 60 min incubation on ice with intermittent vortexing. The protein-containing supernatant was collected in a fresh sterile microcentrifuge tube and used for WB. Serum-derived from 3^rd^ trimester healthy pregnant women was used as a positive control.

### 2.4 Protein isolation and western blot analysis

Radioimmunoprecipitation assay lysis buffer (RIPA) containing a protease inhibitor cocktail (HiMedia; #ML051) was used to homogenise placental tissues (~40 mg). Homogenised lysates were centrifuged at 15000 rpm for 20 min at 4 °C. The protein was quantified using Folin-Lowry’s method. 40 µg of protein was loaded in each well and was resolved under reducing conditions via 8% v/v SDS-PAGE. The proteins were transferred onto a nitrocellulose membrane (Bio-Rad). The membrane was then blocked for 45 min at room temperature using 2% w/v bovine serum albumin (BSA; Sigma Aldrich; #A2153) in TBS containing 0.1% v/v Tween-20 (SRL; #65296) (TBST) for properdin, while 3% non-fat dry milk powder (SRL; #28582) in TBST was used in the case of C3, C5 and β-actin. The membrane was probed with mouse anti-human properdin monoclonal antibody (#HYB 3904, 0.9mg/ml in PBS, 1:2000 dilution in 1% BSA in TBST), rabbit anti-C3 polyclonal antibody (ABclonal A16781, 1:2000 dilution in TBST) and rabbit anti-C5/C5a polyclonal antibody (ABclonal A8104, 1:700 dilution in TBST), respectively, at 4 °C overnight. Rabbit polyclonal antibody against human β-actin (Abcam, ab-16039, 1:2000 dilution in TBS) was used as loading control. Membranes were washed in TBST and probed with HRP-conjugated goat anti-mouse IgG (H+L) (ABclonal AS003, 1:4000 dilution in 1% BSA in TBST) for properdin, and HRP-conjugated goat anti-rabbit IgG (H+L) (ABclonal AS014, 1:10000 dilution in TBST for C3, and 1:2000 dilution in TBST for C5) for 2h at room temperature followed by washing. Finally, immobilon western blotting substrate (Milipore; #WBLUF0100) was used to detect the protein bands. The WB experiments were repeated three times and representative images were selected for presentation. Intensity was measured using ChemiDocTM MP Imaging System (Bio-Rad) and densitometric analysis was carried out using ImageJ software.

For WB using circulating placental exosomes, approximately 50 µg of protein extracted from serum and circulating placental exosomes of PE and CTRL pregnant women were separated on 12% SDS-PAGE gradient gel at 100V (90 min). The gel was transferred to polyvinylidene difluoride (PVDF) membrane (Pall Life Sciences). After blocking the membrane with 3% BSA in PBS-T (PBS containing 0.1% Tween-20), the membrane was incubated overnight with mouse anti-human properdin primary antibody (1:1000; #HYB 3904). Next day, the membrane was washed with PBS-T and PBS for 5 min. This followed incubation with goat anti-mouse HRP-conjugate (1:5000; Invitrogen; #31430). After washing with PBS-T and PBS of 5 min each, the blot was developed. The immunoblot experiments were repeated three times and representative images were selected. The relative protein levels of properdin in individual EOPE and control placental exosome lysates were estimated by densitometric analysis using ImageJ software. CD9 (1:500; Abcam; #ab58989) was used as a loading control for the densitometric analysis, which is a standard marker of exosomes.

Placental STBMs, isolated from CTRL or PE patients, were prepared using a dual-placental perfusion system, as previously described (33, 48). 20 μg of STBMs, diluted in 2x Laemmli buffer, were subjected to a 10% v/v SDS-PAGE under reducing conditions. Proteins were transferred to a nitrocellulose membrane using the wet system of Mini Blot Module (Thermo Fisher Scientific). After blocking for 1h with 5% skimmed milk in TBST, the membranes were incubated overnight with primary antibodies at 4 °C: anti-human properdin (1:1000; Millipore; #MABF2127), and anti-β-actin (1:1000 dilution; Santa Cruz; #sc-8432). The following day, the membrane was incubated with anti-mouse LI-COR IRDye 800CW and anti-mouse LI-COR IRDye 680CW (1:10, 000 dilution; LICOR Biosciences, Lincoln, NE, USA) for 1h at room temperature. The fluorescence intensity was acquired in the Odyssey^®^ CLx near-infrared scanner (LI‐COR Biosciences, Lincoln, NE, USA) and normalized for anti-β-actin. Image acquisition, processing and data analysis were performed via Image Studio Ver 5.2 (LICOR Biosciences).

### 2.5 Immunohistochemical analysis

PE and CTRL placental cotyledons were sampled in the marginal zone bordering the yolk sac, maximum 4 h after birth, and the sample was washed with saline to remove blood before being fixed in 10% v/v buffered formalin, embedded in paraffin, and stored at 4 °C. Tissue sections of 3-4 µm were deparaffinized with xylene and rehydrated in decreasing concentrations of ethanol (100%, 95%, 70%) and dH_2_O. Antigen unmasking was performed using citrate buffer (pH 6) for 20 min at 98 °C in a thermostatic bath. After neutralization of the endogenous peroxidase activity with 3% v/v H_2_O_2_ for 5 min, the sections were incubated in PBS + 2% w/v BSA for 30 min to block non-specific binding. Next, mouse anti-human properdin monoclonal antibody (1:50; Santa Cruz) was incubated at 4 °C. Staining was revealed via anti-mouse horseradish peroxidase (HRP)-conjugate (1:500 dilution, Sigma), incubated for 30 min at room temperature, and the substrate, 3-amino-9-ethylcarbazole (AEC, Vector Laboratories). Sections were counterstained with Mayer Haematoxylin (DiaPath, Italy) and examined under a Leica DM 2000 optical microscope. Kidney tissue was stained as a positive control. Images were collected using a Leica DFC 7000 T digital camera (Leica Microsystems, Wetzlar, Germany). To quantify the staining, H score was calculated via ImageJ.

### 2.6 Immunofluorescence microscopy

Term placental tissues from PE and CTRL groups were fixed in 10% v/v buffered formalin, paraffin-embedded and stored at 4 °C. Tissue sections of 3-4 µm were deparaffinized with xylene for 30 mins twice and rehydrated with decreasing concentrations of ethanol (100%, 95%, 70%, 50% and 30%) for 10 min each. The antigen unmasking was performed using citrate buffer, pH 6 for 2 min at 98°C in a microwave. Sections were then washed five times in PBS buffer, pH 7.4 and incubated with PBS + 10% w/v BSA at 4 °C for 1.5 h to block non-specific binding. Next, mouse anti-human properdin monoclonal antibody (HYB 3904; 1:100) and rabbit polyclonal antibody against human cleaved caspase 3 (Cell Signaling Technology; 1:400 dilution) in 2% BSA in PBS were added to the sections, and incubated overnight at 4°C. Next day, after washing with PBS five times, sections were incubated overnight with goat anti-mouse Alexa FluorTM 488 (Thermo Fisher Scientific; 1:500 dilution) and chicken anti-rabbit Alexa FluorTM 647 (Thermo Fisher Scientific; 1:500 dilution) conjugates, respectively, at 4°C for 2 h, followed by 4’, 6-diamidino-2-phenylindole (DAPI) staining for 10 min in dark at room temperature. Following two 5-min washes with PBS, the sections were mounted with Dibutylphthalate polystyrene xylene (DPX) (Merck). Images were captured using fluorescence microscope (ZEISS Scope.A1).

### 2.7 Transmission electron microscopy

PE placental tissues were fixed in 2.5% v/v glutaraldehyde (TAAB Laboratories, UK, #G002) and 2% v/v paraformaldehyde (Thermo Fisher) in 0.1 M phosphate buffer (pH 7.3) for 2 h at 4 °C. After washing in PBS, sections were fixed in 1% osmium tetroxide (TAAB Laboratories, UK, #O001) for 1 h and dehydrated in acetone (30%-50%-70%-80%-90%-95% twice for 10 min at 4°C). The samples were embedded in araldite CY212 (TAAB Laboratories, UK) and then polymerized at 60 °C for 72 h. Ultra-thin sections (60–70 nm) were stained with aqueous uranyl acetate and alkaline lead citrate (LADD Research, USA). The sections were observed under a Tecnai G2–20 S-twin transmission electron microscope (Fei, Eindhoven, Netherlands) at a magnification range of 2000–10000X. TEM study was carried out at the All India Institute of Medical Sciences, New Delhi, India.

### 2.8 Enzyme-linked immunosorbent assay

For measuring the levels of properdin in PE and CTRL sera, human complement properdin ELISA kit (Hycult Biotech; # HK334) was used; instructions provided by the manufacturer were followed for the assay. The plate was read by the PowerWave X Microplate Reader (Bio-Tek Instruments) spectrophotometer at 450 nm.

### 2.9 Statistical analysis

For statistical analysis, the non-parametric Mann-Whitney U test was used to compare two groups (CTRL and PE) for IHC, WB (placental tissues, exosomes, and STBM analyses), and RT-qPCR. Matched data comparing PE *vs* healthy CTRL were analysed through paired Wilcoxon signed-rank test. Results were expressed as mean ± standard deviations. All statistical analyses were performed using GraphPad Prism software 9.0 (GraphPad Software Inc., USA). Results were considered statistically significant for all analyses when *p <0.05, **p <0.01.

## 3 Results

### 3.1 Increased local production of properdin in PE placentae is linked to augmented C3 and C5 expression levels compared to healthy placentae (CTRL)

**Figure 1:**
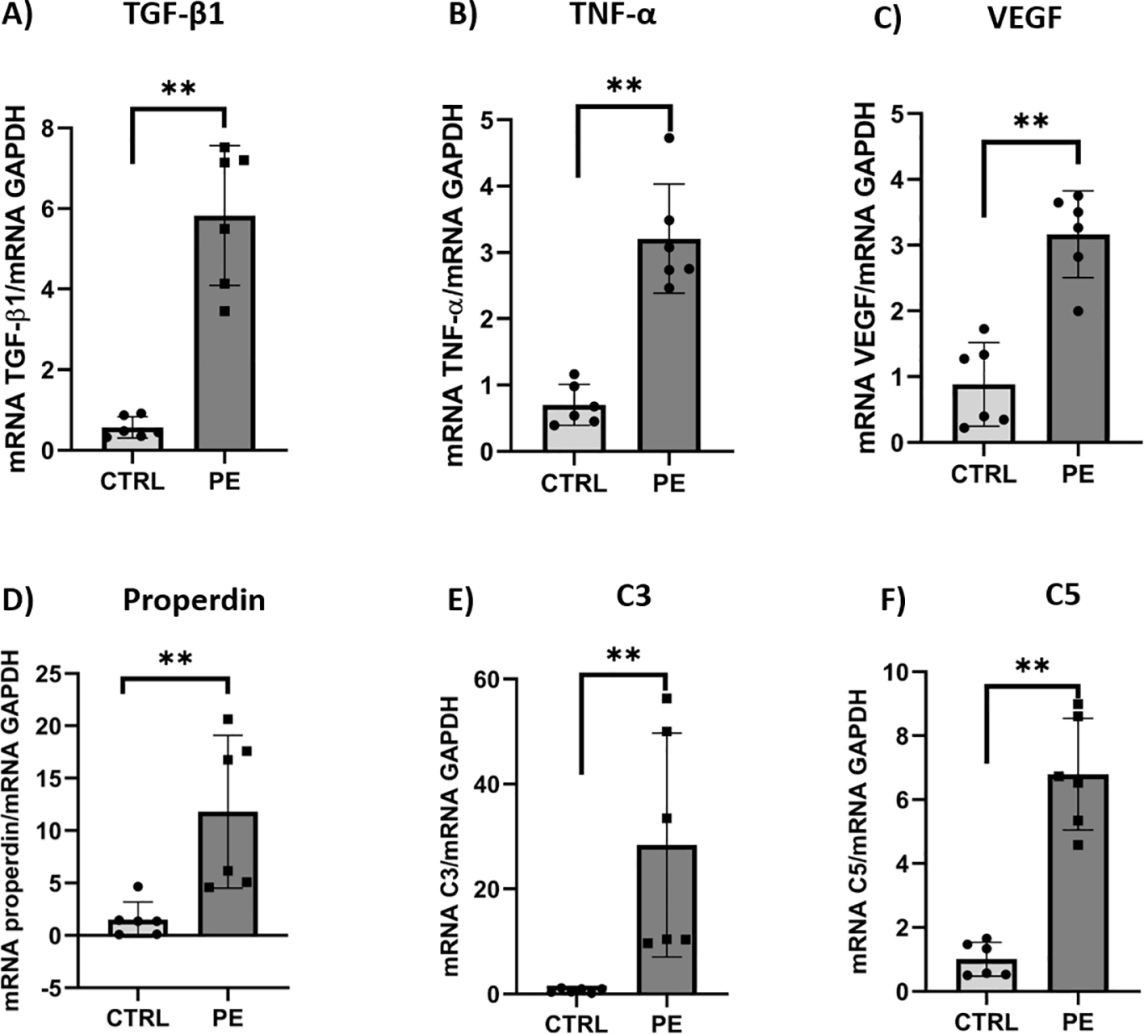
mRNA expression of **(A)** TGF-β1, **(B)** TNF-α, **(C)** VEGF, **(D)** properdin, **(E)** C3 and **(F)** C5 in the third trimester placental tissue of preeclampsia (PE, n = 6) compared to normal pregnancy (CTRL, n = 6) through RT-qPCR. GAPDH was used as a housekeeping gene to normalize gene expression results. The mRNA expression of all transcripts were significantly higher in PE placenta compared to placenta of healthy mothers. Data are expressed as mean ± SD of two independent experiments performed in triplicate. Significance was compared as PE vs CTRL and marked with asterisk **p < 0.01, (non-parametric Mann-Whitney U test).

As little information is available regarding properdin expression in placentae, we initially focused on mRNA expression in PE placentae compared with CTRL tissues *via* RT-qPCR. To assess the inflammatory condition of the PE placenta, we first examined the mRNA expression levels of three key inflammatory markers (*i.e.*, TGF-β1, TNF-α and VEGF), which are commonly dysregulated in PE. The PE placentae, belonging to our cohort of patients, showed significantly higher levels of mRNA expression of TGF-β1 (6-fold, **Figure 1A**), TNF-α (3-fold, **Figure 1B**), and VEGF (3-fold) (**Figure 1C**), compared to CTRL. The mRNA levels of properdin in the same placentae were nearly 10-fold higher in PE compared to CTRL (**Figure 1D**). Since properdin is the only known positive regulator of the complement alternative pathway, we also analyzed the mRNA expression of C3 and C5 genes, which were found to be significantly over-expressed in PE placentae compared to the CTRL (**Figures 1E, F**).

**Figure 2:**
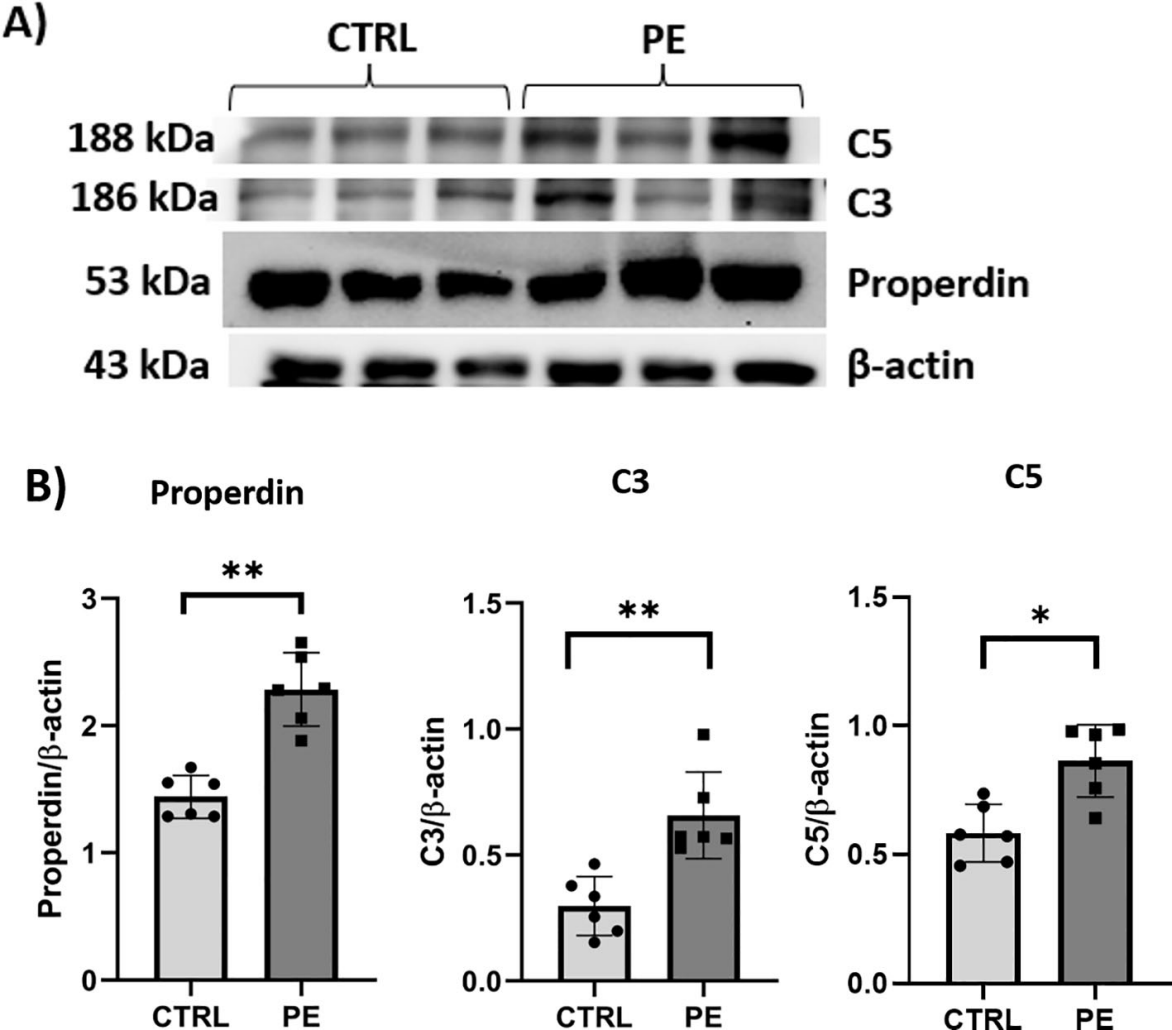
Protein expression of properdin, C3, and C5 in placental tissue of PE compared to CTRL pregnancies through WB analysis. **(A)** Representative image of the blot for C3, C5, and properdin, where β-actin was used as the loading control. **(B)** Quantitative analysis of the band intensity of protein expression of properdin, C3, and C5, normalized with the loading control (β-actin). The graph represents mean ± SD values of *n* = 6 (each for PE and CTRL) placental tissues (non-parametric Mann-Whitney U test, *p < 0.05 and **p < 0.01). The protein expression of properdin, C3 and C5 was significantly higher in PE placenta compared to CTRL.

Secondly, we analysed them at the protein level by WB. Our results confirmed what was observed for the properdin, C3, and C5 transcripts (**Figure 2**). The WB analysis revealed significantly higher levels of these complement proteins in PE placental tissues compared to CTRL (**Figures 2A, B**). Thus, properdin mRNA and protein levels remain significantly higher in PE placenta compared to CTRL (**Figures 1D**, **2A, B**), along with the complement proteins, C3 and C5 (**Figures 1E, F**, **2A, B**).

### 3.2 Localization of properdin in term placental tissues of both PE and CTRL women

**Figure 3:**
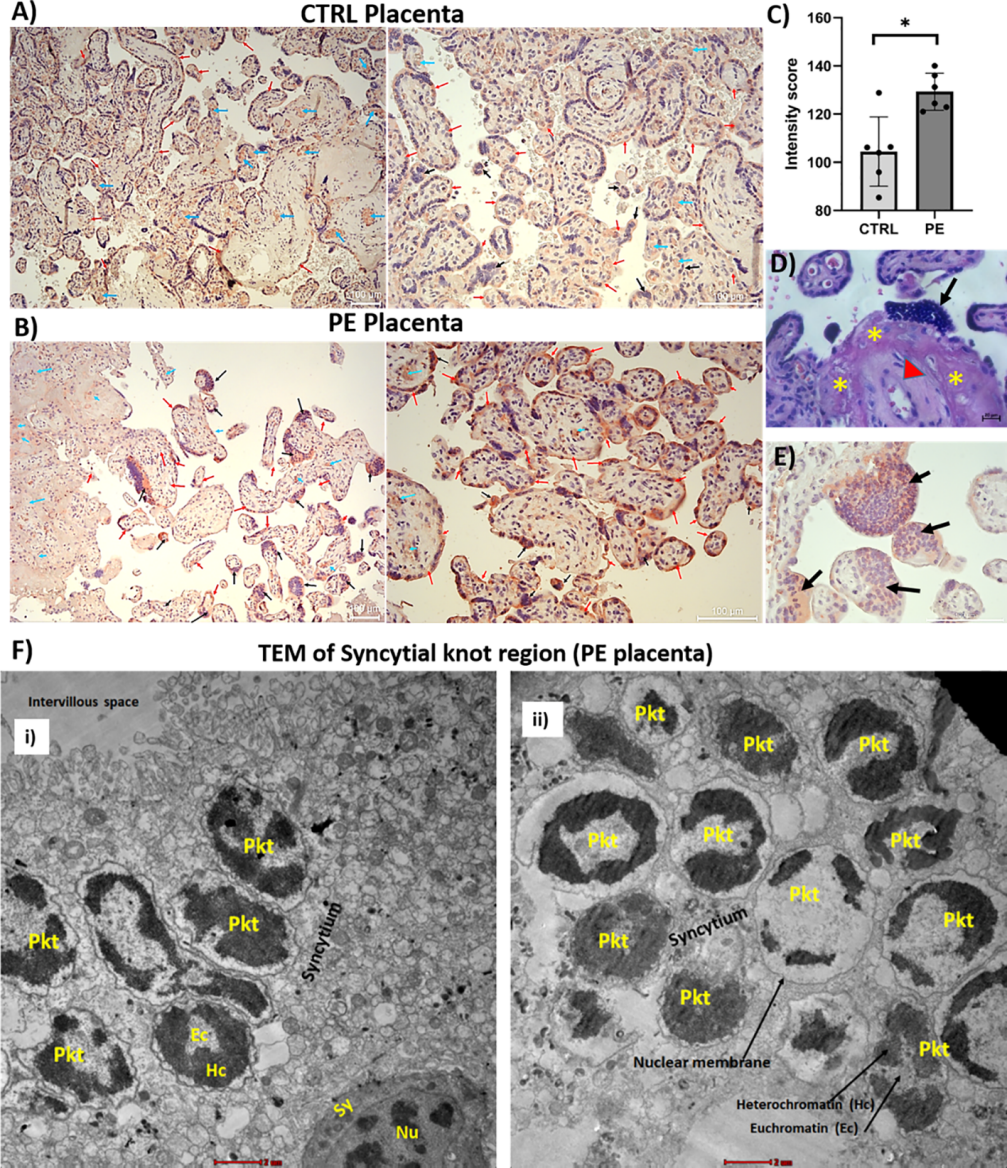
Bright field and electron microscopic images of placental tissues (PE and CTRL). Localization of properdin in placental tissues from **(A)** CTRL (upper panel) and **(B)** PE (lower panel). Placental samples of CTRL and PE pregnancies were stained with anti-properdin antibody. Properdin staining was detected in all CTRL and PE placental tissues, being localized in fetal villi endothelium (blue arrows) and in the syncytiotrophoblast monolayer (red arrow), although the intensity varied. In PE placenta, properdin was detected more prominently in the syncytial knots (black arrow). Staining was detected *via* 3-amino-9-ethylcarbazole (AEC) substrate chromogen. Nuclei were stained with Mayer’s Hematoxylin. Scale bars, 100 µm (left pictures’ original magnification 10X; right pictures’ original magnification 20X). **(C)** Quantitation of IHC staining (6 images analysed for CTRL *vs* PE). For each slide, three different visual fields of the microscope were analysed giving a score as described in Materials and Methods section. For statistical analysis non-parametric Mann-Whitney test was used. **(D)** Bright field image of haematoxylin and eosin (H&E) stained PE placenta, original magnification 40X (scale bar, 20 µm). Deep haematoxylin nuclear staining was observed at the placental knot regions (black arrow). The tissues also showed fibrinoid deposition (asterisks) and thickened blood capillaries (red arrowhead). **(E)** High resolution image of the syncytial knots in PE placenta stained deeply with anti-properdin antibody (black arrow). Scale bars, 100 µm, original magnification 40X. **(F)** Ultrastructure of the syncytial knot region under TEM (i and ii), scale bar is 2 µm. The image showed presence of several pyknotic syncytial nuclei (Pkt) mostly surrounded by its nuclear membrane at the PE placental knot region. The nuclei seem to be in their advanced stages of degeneration (apoptosis/necrosis). *p < 0.05.

To investigate the distribution of properdin in placental tissues from both PE and normal pregnancies, we assessed the properdin levels in term placental tissues *via* IHC. Staining for properdin was detected in both CTRL and PE placenta, particularly within the fetal villi endothelium (indicated by the blue arrows) and in the syncytiotrophoblast monolayer (red arrows) (**Figures 3A, B**, **Supplementary Figures 2A, B**). However, the intensity of properdin staining was comparatively lower in CTRL *vs* PE samples (**Figure 3C**). The PE placentae showed quite a high level of properdin in the syncytial knots (black arrows), which were high in number, and also throughout the syncytiotrophoblast monolayer (red arrows) across almost all samples (**Figure 3B**; **Supplementary Figure 2B**).

### 3.3 Presence of pyknotic nuclei inside PE syncytial knots

Since properdin was highly concentrated in the syncytial knots of PE placentae, we decided to carry out TEM study of this region. The PE placenta exhibited intense haematoxylin nuclear staining at the placental knot regions (black arrow) under a bright-field microscope (**Figure 3D**). A high-resolution image of the syncytial knots in PE placenta stained deep red with anti-properdin antibody (black arrows), which is shown in **Figure 3E**. The TEM images revealed the presence of several pyknotic nuclei inside the syncytial knot structures in the PE placenta, which seems to be mostly at their late degenerative state (**Figure 3F**). The small round pyknotic nuclei were present in the syncytium with dense peripheral condensed heterochromatin. The heterochromatin was abundant compared to euchromatin and was surrounded by nuclear membrane, leaving little space in between the membranes.

### 3.4 Higher expression of properdin and cleaved caspase-3 inside PE syncytial knots

**Figure 4:**
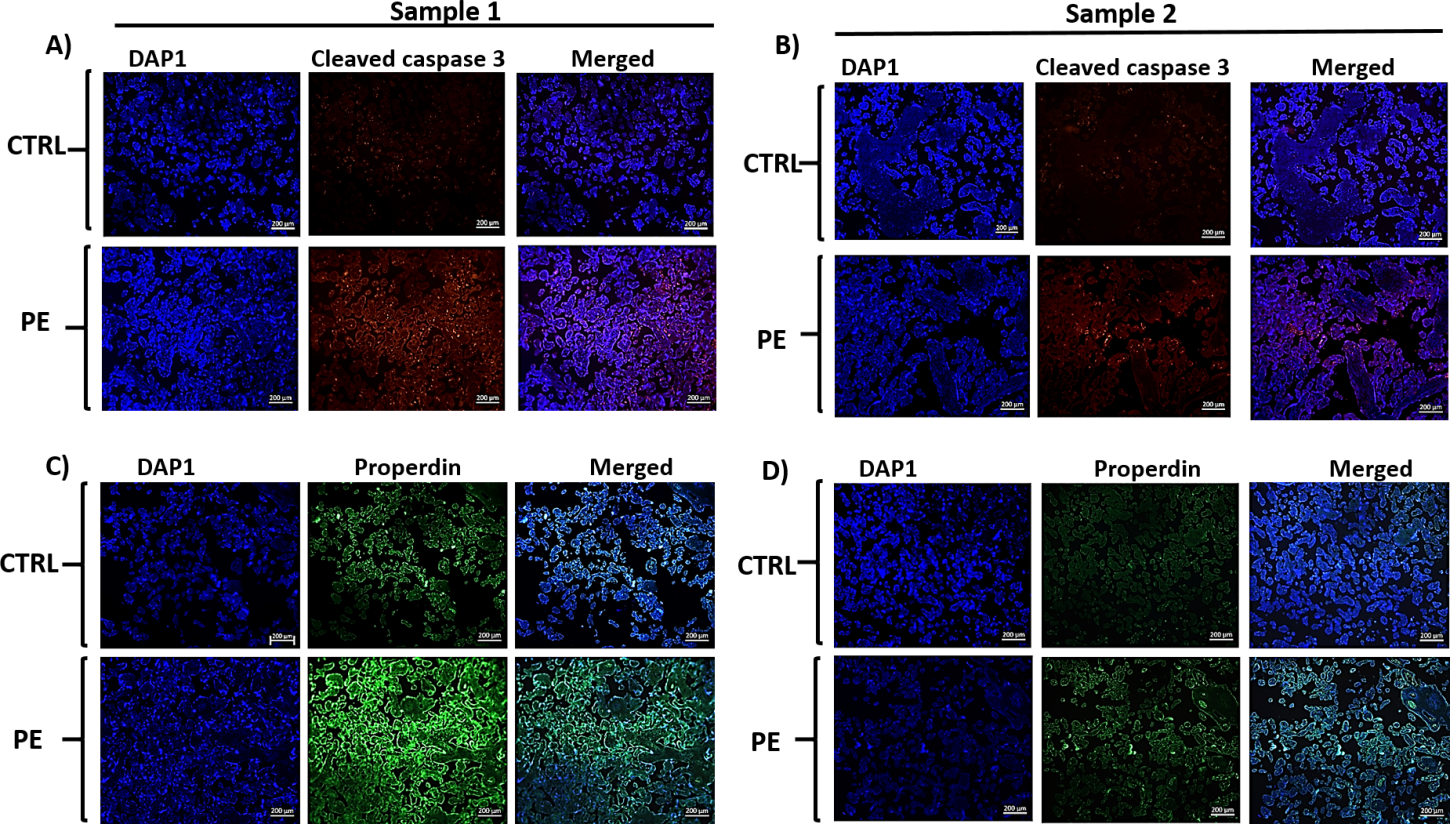
Immunofluorescence images of tissue sections obtained from CTRL and PE placental tissue samples. Two different samples (sample 1 and sample 2) were stained with anti-cleaved caspase 3 antibody **(A, B)** red colour, or anti-properdin antibody **(C, D)** green colour. All the tissues were counterstained with DAPI (blue colour). Images were observed and photographed under fluorescence microscope (ZEISS Scope.A1) under 10X magnification, the scale bar mentioned is of 100µm. All the PE placental samples showed high level of fluorescence emission for cleaved caspase 3 and properdin compared to CTRL.

**Figure 5:**
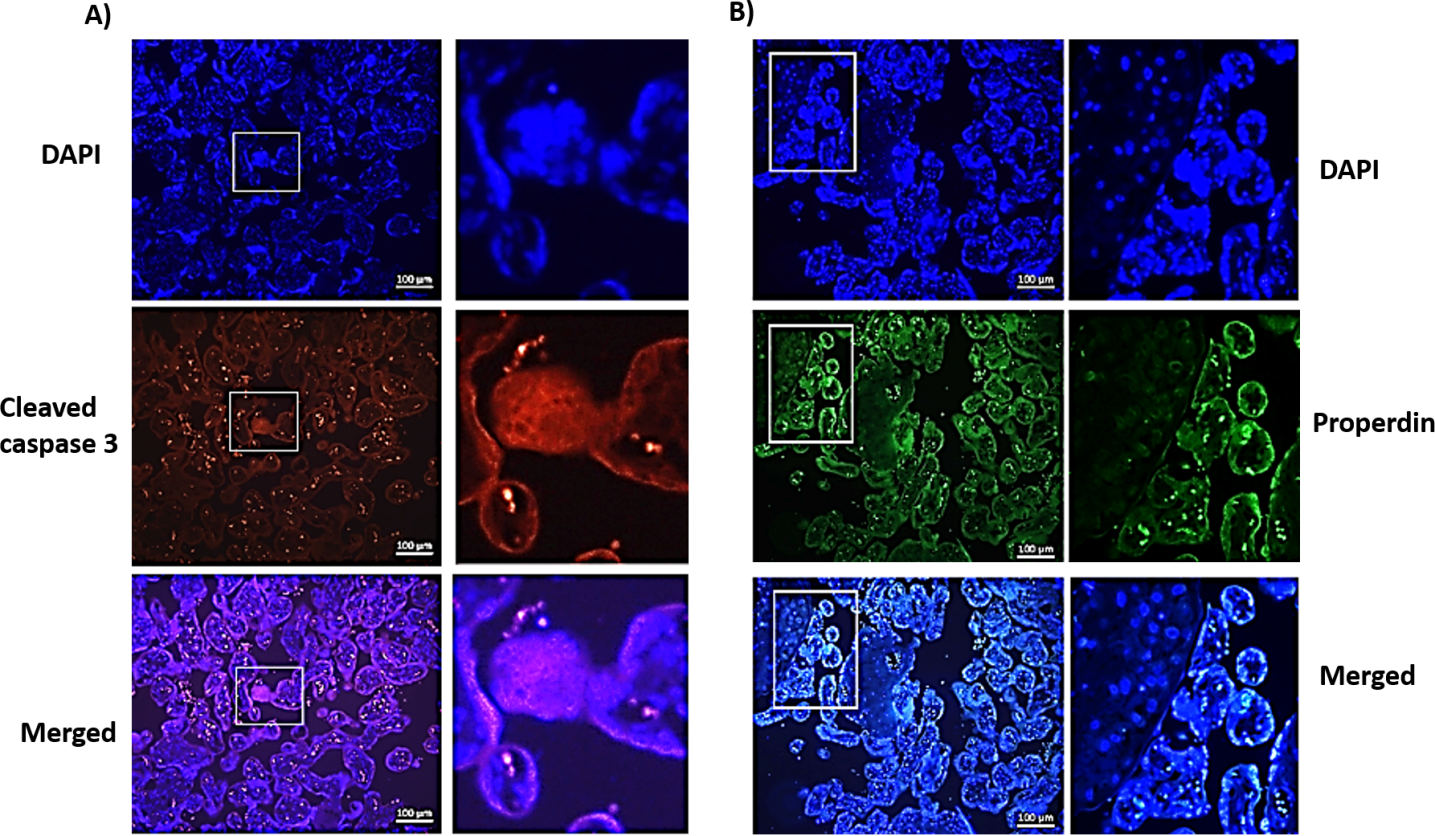
Immunofluorescence images of PE placenta, **(A)** cleaved caspase 3 (red colour) and **(B)** properdin (green colour). In each case left panel shows images with 10x magnification (scale bar is of 100µm) and right panel shows magnified images of the syncytial knot region. The tissue sections were counter stained with DAPI (blue colour) and images were observed and photographed under fluorescence microscope (ZEISS Scope.A1). Both cleaved caspase 3 and properdin protein were present in the syncytial knot region which also stained high DAPI staining.

Pyknotic nuclei can form during apoptosis as well as necrosis, and evidence of both of these processes was observed in the PE placenta (15, 49–51). Thus, to confirm the apoptotic characteristics of the nuclei in PE syncytial knots, we investigated the expression of cleaved caspase 3, an apoptotic protein marker. Although the data obtained were not quantitative, the PE placentae showed strong immunofluorescence with anti-cleaved caspase 3 antibody (red colour) compared to CTRL placentae (**Figures 4A, B**). The serial sections of PE placenta also exhibited higher immunofluorescence for anti-properdin (green colour) compared to CTRL placental sections (**Figures 4C, D**). High magnification images showed the presence of both cleaved caspase 3 (**Figure 5A**) and properdin proteins (**Figure 5B**) in the syncytial knot regions of the PE placenta, which also revealed high nuclear staining (DAPI).

### 3.5 Low properdin expression in the serum in PE patients compared to CTRL

**Figure 6:**
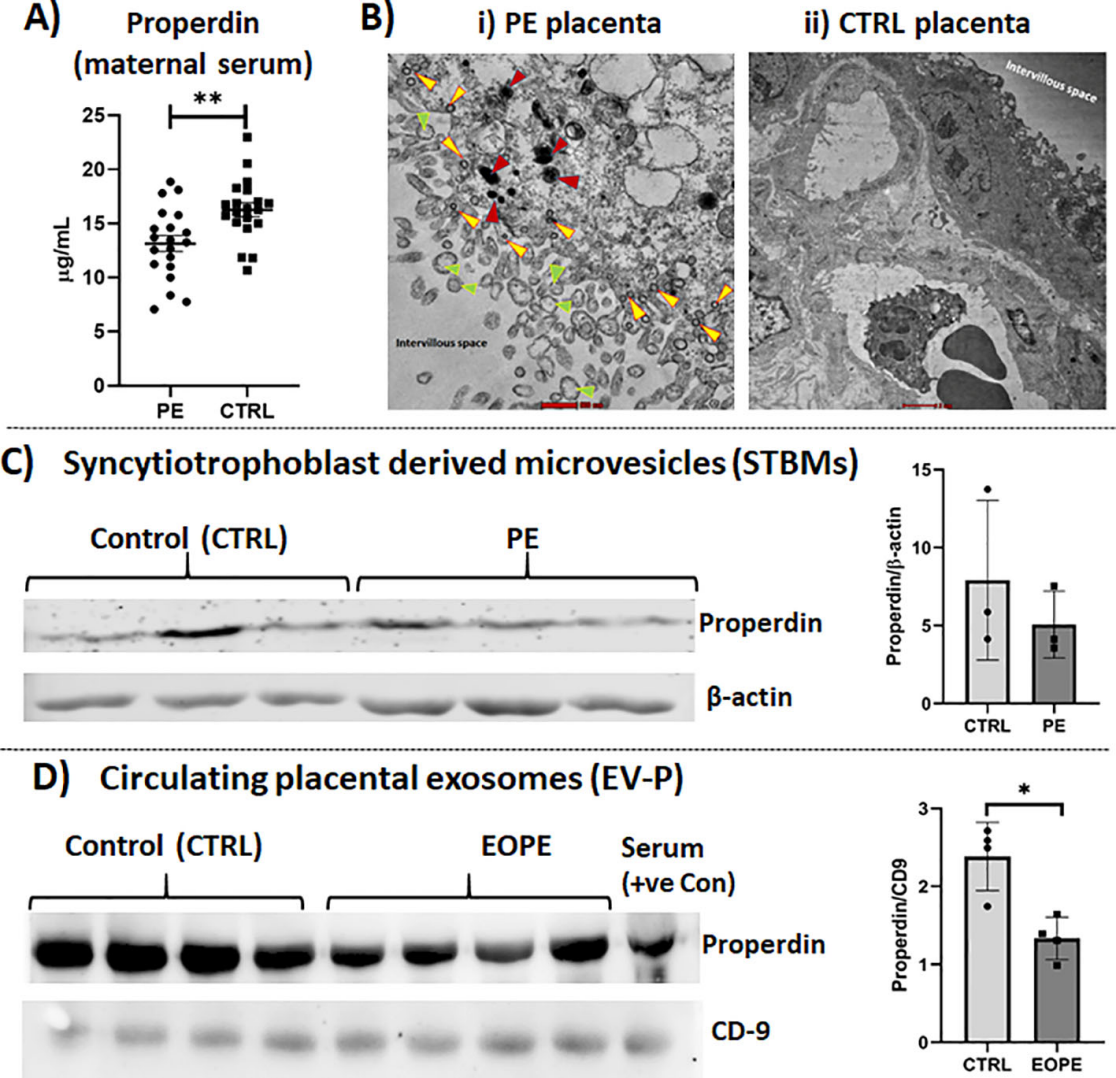
**(A)** Measurement by ELISA of circulating properdin levels in sera of CTRL and PE women, collected during 3^rd^ trimester. Lower levels of properdin were observed in PE pregnancies compared to CTRL, showing a highly significant difference (*n* = 20 PE vs *n* = 20 CTRL). **p < 0.01 (paired Wilcoxon signed-rank test). **(B)** TEM image of: i) PE placenta showing syncytiotrophoblast fragments as extracellular vesicles of varied sizes, cell debris, apoptotic nuclei, possibly released towards the intervillous space. The exosomes are marked with yellow arrowheads, STBMs with green arrowheads, and pyknotic nuclei with red arrowheads (scale 500 nm). ii) CTRL placenta showing almost an intact syncytiotrophoblast layer (outer layer facing intervillous space) and cytotrophoblast layer lying underneath. **(C)** Evaluation of properdin deposition on placental STBMs *via* Western blot (WB). STBMs derived from PE (*n* = 3) and CTRL (*n* = 3) were probed with anti-properdin antibody. β-actin was used to normalize the results. **(D)** Evaluation of properdin levels in circulating placental exosomes of 3^rd^ trimester *via* WB. Properdin level was significantly decreased in PE-derived placental exosomes compared to CTRL. Proteins extracted from circulating placental exosomes from PE (*n* = 4) and CTRL (*n* = 4) women were probed with mouse anti-human properdin primary antibody. The WB data is represented as mean ± SEM. CD9 was used to normalize the results. *p < 0.05 (Mann-Whitney U test).

Having noted high level of properdin mRNA and protein in the PE placental tissue, we sought to estimate the level of properdin protein in the PE serum by ELISA. Strikingly, properdin levels in the PE serum were significantly lower compared to healthy mothers (CTRL) (**Figure 6A**). Indeed, the properdin levels ranged between 7.08 and 18.87 µg/ml in PE sera (mean 13.14 ± 3.3), and 10.68 and 22.99 µg/ml (mean 16.26 ± 2.9) in healthy CTRL.

### 3.6 Presence of properdin in the placental microvesicles at lower level in the PE placental STBMs and circulating placental exosomes compared to CTRL

Placental extracellular vesicles are increasingly recognized as key mediators in the development of PE, even though the underlying mechanisms are not fully understood. Given that both placental STBMs and circulating placental exosomes were found to be significantly increased in PE mothers in our earlier studies, we decided to examine whether these microvesicles were differentially loaded with properdin in PE and CTRL. TEM images of the PE placenta revealed a substantial shedding of syncytiotrophoblast fragments as extracellular vesicles of varied sizes, along with cell debris and apoptotic nuclei, in the intervillous space. The exosomes (yellow arrowheads), STBMs (green arrowheads), and pyknotic nuclei (red arrowheads) were observed in the PE placenta exposed towards the intervillous space (**Figure 6B**i). CTRL placenta showed an almost intact syncytiotrophoblast layer (outer layer facing intervillous space) and cytotrophoblast layer lying underneath (**Figure 6B**ii). The level of properdin protein in STBMs from PE mothers compared to CTRL showed no significant difference in WB analysis (**Figure 6C**). However, the properdin protein levels in circulating placental exosomes from PE mothers were significantly lower compared to those in CTRL (**Figure 6D**).

## 4 Discussion

Properdin upregulates the complement alternative pathway, playing a crucial role in the body’s natural resistance to pathogens (52). In human, properdin is present in a very low concentration, ranging between 5 and 25 µg/ml in plasma, to ensure an optimal level of the complement alternative pathway activation (41, 45, 46, 53–56). The ratio of properdin polymers exists at a natural distribution of 26:54:20 (P2:P3:P4), suggesting a controlled steady-state of complement activation (54, 57, 58). Properdin enhances complement activation by binding to C3b, which prolongs the half-life of C3 convertase by 5 to 10 times, thereby acting as an initiator and enhancer of the alternative pathway (42, 59). Infiltrating leukocytes at the fetal-maternal interface can secrete properdin in response to pro-inflammatory signals, increasing its concentration in the local tissue environment.

In this study, together with an elevated expression of inflammatory markers (*i.e.*, TGF-β1, TNF-α, and VEGF) in PE placentae, we observed that the mRNA expression of properdin was several fold higher than in healthy mothers. The mRNA levels of C3 and C5 were also significantly higher in the PE placenta. These findings were confirmed also at the protein level, suggesting a significantly higher level of properdin in the PE placenta, along with C3 and C5 proteins.

To investigate whether properdin is synthesized locally, we examined its distribution in the placental tissue. Properdin was found in the syncytiotrophoblast layer, in the fetal villi endothelium, as well as in the syncytial knots in both PE and CTRL placentae, although properdin staining was stronger in PE placentae. However, Buurma et al. have shown the presence of properdin only in the fetal villi endothelial cells, in PE as well as in healthy placenta (29). The PE placenta showed significantly enhanced C3 level compared to healthy mothers (60). Two different studies have shown the presence of high C4d deposition in the PE placenta (29, 60, 61). Excessive C3d and C9 (but not C1q) deposition in the PE placenta has also been observed (61). Belmonte et al. performed a comprehensive overview of complement activation in PE placentae (40), demonstrating the pivotal role of the complement lectin pathway, activated, not by MBL (62) but by ficolin-3. Properdin may play a key role in sustaining the auto-amplifying loop induced by the complement alternative pathway, following activation of the lectin pathway mediated by preeclamptic hypoxia and oxidative stress. We have shown recently that factor H (FH), a negative regulator of complement alternative pathway, is almost absent in the PE placenta (63). This suggests that the complement system is dysregulated in the PE placenta, potentially due to excessive complement activation or insufficient inhibitory regulation, or a combination of both. Unlike most complement proteins that are mostly synthesized in the liver, the source of properdin can be extrahepatic (41, 64). The significant presence of properdin in the PE placenta suggests that it could not only originate from infiltrating immune cells but may also be locally synthesized by placental cells.

It is likely that complement activation is not a direct cause of PE onset but rather a key player in the exponential progression and development of placental damage. We believe that hypoxic stress induces activation of the lectin pathway and, thus, the activation of the amplification-loop by the alternative pathway. The resulting inflammatory state also upregulates the local expression of some key components, such as properdin, which contribute to tissue damage and systemic endothelial dysfunction. Furthermore, the lectin pathway can also recognize the host DNA exposed on apoptotic cells. Collectin-12 may activate the alternative pathway in its soluble form in conjunction with properdin (65).

Our IHC results revealed that the PE placenta exhibits an elevated presence of syncytial knots, containing very high amounts of properdin. Syncytiotrophoblast layer of the PE placenta also stained deeply with anti-properdin antibody compared to CTRL placenta. Hematoxylin and eosin staining took heavy nuclear stain in the syncytial knot region. TEM images of the PE placental knot region showed several aggregates of denser pyknotic syncytial nuclei with condensed chromatin. Sharp et al. also observed similar apoptotic features in the syncytiotrophoblast nuclei undergoing pyknosis, and confirmed the presence of cleaved DNA in the PE placenta (66). Apoptosis, a physiological phenomenon observed during placental remodeling, can increase further in pathological conditions, particularly in PE placenta (67–72). Our present study showed high expression of cleaved caspase 3, an apoptotic marker, in the PE placenta compared to CTRL. Others have documented significantly increased levels of caspase-3 in the trophoblast villous region of PE placenta (73). Our higher magnification images showed co-localization of cleaved caspase 3 with the nuclear material in the PE syncytial knot region. In PE, the expression of Smac, a caspase inhibitor, is known to be significantly increased compared to normal pregnancy (69).

Pyknosis can occur in both apoptosis and necrosis (49). Xu et al. observed that, similar to C1q and MBL, properdin binds to cells in their late apoptotic and necrotic stages, where exposed DNA acts as a ligand for properdin. This induces amplification of complement alternative pathway that is independent of C3b deposition (74). Our immunofluorescence images showed the co-localization of properdin with syncytial nuclei in the PE knot region. This association of properdin with the apoptotic DNA, acting as a pattern recognition molecule, is either to flag for macrophage-mediated clearance, or for recruiting C3b. We believe that the high amount of properdin in placenta is likely to recruit C3b, stabilize the inherently labile C3bBb complex, and contribute to the local complement activation. A limitation of this study is a lack of demonstration about the deposition of activated complement factors in the placental samples investigated for properdin.

The elevated level of Bb in both maternal plasma and umbilical venous plasma in PE subjects also indicates complement alternative pathway activation in PE (75). Moreover, we also showed here high amounts of C3 and C5 in PE placenta. Additionally, neutrophil infiltration is also heightened in PE placenta with increased incidence of NETosis (76). The degranulation enzyme myeloperoxidase, released from activated neutrophils, can also direct properdin-mediated alternative pathway (77). As neutrophils are one of the major sources of properdin (78), whether the large number of infiltrated neutrophils in the PE placenta contribute to the high amount of properdin needs further investigation.

In this study, even though we observed higher properdin levels in the PE placenta, the concentration of properdin was found to be lower in the serum of PE mothers. Throughout all stages of pregnancy, serum properdin levels have been found to be lower than those of non-pregnant women in the same age group (52). Moreover, the serum level of properdin is significantly lower during the 1^st^ and the 2^nd^ trimesters compared to the 3^rd^ trimester of pregnancy (52). Our findings on the properdin levels are consistent with previous studies. Blakey et al. found similar patterns analyzing two different cohorts of PE patients compared to healthy pregnant women: the properdin concentration in both maternal and umbilical cord blood of PE samples was significantly lower compared to controls (61). Interestingly, the serum level of properdin tends to be lower in pathological conditions than in healthy individuals. Stover et al. reported that the level of properdin in the normal sera is 18.4 ± 5.5 µg/ml; in critically ill patients with sepsis, properdin level goes down with a mean value of 9.0 ± 7.6 µg/ml in the 1^st^ day of ICU, further reducing to 6.8 ± 5.2 µg/ml (0 to 21.2 µg/ml) in case of non-survivors (56). In SLE, properdin level was around 17.06 µg/ml, while it remained 25 ± 4 µg/ml in healthy subjects (45). The reduction of properdin in pathological conditions may be attributed to the utilization/consumption of properdin in the affected tissues. However, it is important to note that these reduced levels could also be due to an altered synthesis, a modified compartmentalization, or changes in the polymeric states of properdin.

The predominant theory regarding the development of PE recognises the “two-stage model.” In the initial stage, there is a reduction in placental perfusion coupled with hypoxia and premature ageing of the placenta, which induces stress on the syncytiotrophoblast. This stress subsequently leads to the second stage, characterized by oxidative stress, mitochondrial dysfunction, and apoptosis. These processes result in the release of various dysregulated factors from the syncytiotrophoblast, including syncytial knots, DNA fragments, small extracellular vesicles, apoptotic bodies, reactive oxygen species, and pro-inflammatory cytokines (79). Our TEM images of PE placentae revealed a large number of vesicles being shed into the intravillous space. These vesicles are similar in size to exosomes (*i.e.*, 50–150 nm) and STBMs (*i.e.*, 100–1000 nm). Placenta-derived circulating exosomes have been found to play immunomodulatory roles during pregnancy; they also increase in PE as compared to a healthy pregnancy, indicating an altered functionality of microvesicles during PE (18, 19). STBMs are significantly elevated in the plasma of PE women (16, 80). STBMs in PE were found to be pro-inflammatory and anti-angiogenic with altered surface glycan (16). To understand the association of properdin with the placental microvesicles, we isolated placental STBMs and circulating placental exosomes from PE and CTRL. With regard to STBMs, results were inconclusive. The level of properdin in the circulating placental exosomes in PE mothers were significantly low compared to CTRL. It will be important to mention here that in the PE mothers, who were in their early stage, i.e. <34 weeks of gestation, properdin in the circulation maintained a significantly low level, both in the serum and in the circulating placental exosomes. On the contrary, the level of properdin was high in the term end placentae in PE mothers who were in their late gestational age i.e., **>**34 weeks. However, to establish whether the level of properdin gradually increases as the disease progresses, a study on larger cohort and monitoring the properdin level throughout the gestational term is needed.

In conclusion, our study indicates that properdin levels are considerably higher in the placental tissue in PE compared to healthy mothers, along with complement proteins C3 and C5. Given that a rise in properdin level could exacerbate complement activation, whether properdin acts as a trigger for heightened complement activation in PE placenta requires further investigation. In the maternal serum as well as in the microvesicles, properdin was lower in PE mothers. Properdin was localized extensively in the syncytial pyknotic nuclei in the PE placenta, correlating with high cleaved caspase 3 protein. Direct involvement of properdin in PE pathogenesis needs further assessment in terms of the immunomodulatory properties of properdin both in a complement-dependent and complement-independent manner *via* different PE models. In addition, correlating serum properdin levels with complement consumption throughout pregnancy could make properdin a viable biomarker of PE, although given the modest sample sizes and the absence of ROC analysis, sensitivity/specificity estimates, or adjustment for confounders, biomarker-related conclusions need further study. Although several lines of evidence have emerged over the years regarding the role of the complement system in PE (81), our study analyses, for the first time, both the local expression, the serum levels, and the deposition on STBM and exosomes of properdin, demonstrating the connection with apoptotic syncytiotrophoblast knots, which characterize placental damage in PE.

## Data Availability

The raw data supporting the conclusions of this article will be made available by the authors, without undue reservation.

## Supplementary material

SUPPLEMENTARY FIGURE 1 Graphical description of the multiple cohorts of pregnant women from which serum and placental samples were collected.

SUPPLEMENTARY FIGURE 2 IHC analysis of properdin in placental tissues from **(A)** normal (CTRL) and **(B)** preeclamptic (PE) tissues. Both CTRL and PE placental tissues showed properdin staining, being prominently high in the syncytial knots of PE placentae.

## References

[1] Dimitriadis E Rolnik DL Zhou W Estrada-Gutierrez G Koga K Francisco RPV . Pre-eclampsia. Nat Rev Dis Primers. (2023) 9:8. doi: 10.1038/s41572-023-00417-6, PMID: 36797292

[2] Waker CA Kaufman MR Brown TL . Current state of preeclampsia mouse models: approaches, relevance, and standardization. Front Physiol. (2021) 12:681632. doi: 10.3389/fphys.2021.681632, PMID: 34276401 PMC8284253

[3] Yagel S Cohen SM Admati I Skarbianskis N Solt I Zeisel A . Expert review: preeclampsia Type I and Type II. Am J Obstet Gynecol MFM. (2023) 5:101203. doi: 10.1016/j.ajogmf.2023.101203, PMID: 37871693

[4] Hypertension in pregnancy . Report of the american college of obstetricians and gynecologists’ Task force on hypertension in pregnancy. Obstet Gynecol. (2013) 122:1122–31. doi: 10.1097/01.AOG.0000437382.03963.88, PMID: 24150027

[5] Poon LC Shennan A Hyett JA Kapur A Hadar E Divakar H . The International Federation of Gynecology and Obstetrics (FIGO) initiative on pre-eclampsia: A pragmatic guide for first-trimester screening and prevention. Int J Gynaecol Obstet. (2019) 145:1–33. doi: 10.1002/ijgo.12802, PMID: 31111484 PMC6944283

[6] Wallenburg HC Dekker GA Makovitz JW Rotmans P . Low-dose aspirin prevents pregnancy-induced hypertension and pre-eclampsia in angiotensin-sensitive primigravidae. Lancet. (1986) 1:1–3. doi: 10.1016/s0140-6736(86)91891-x, PMID: 2867260

[7] Jung E Romero R Yeo L Gomez-Lopez N Chaemsaithong P Jaovisidha A . The etiology of preeclampsia. Am J Obstet Gynecol. (2022) 226:S844–S66. doi: 10.1016/j.ajog.2021.11.1356, PMID: 35177222 PMC8988238

[8] Pinheiro MB Martins-Filho OA Mota AP Alpoim PN Godoi LC Silveira AC . Severe preeclampsia goes along with a cytokine network disturbance towards a systemic inflammatory state. Cytokine. (2013) 62:165–73. doi: 10.1016/j.cyto.2013.02.027, PMID: 23523008

[9] Liu X Dai LI Zhou R . Association between preeclampsia and the CXC chemokine family (Review). Exp Ther Med. (2015) 9:1572–6. doi: 10.3892/etm.2015.2337, PMID: 26136860 PMC4471657

[10] Aggarwal R Jain AK Mittal P Kohli M Jawanjal P Rath G . Association of pro- and anti-inflammatory cytokines in preeclampsia. J Clin Lab Anal. (2019) 33:e22834. doi: 10.1002/jcla.22834, PMID: 30666720 PMC6528584

[11] Ullah A Zhao J Singla RK Shen B . Pathophysiological impact of CXC and CX3CL1 chemokines in preeclampsia and gestational diabetes mellitus. Front Cell Dev Biol. (2023) 11:1272536. doi: 10.3389/fcell.2023.1272536, PMID: 37928902 PMC10620730

[12] Laresgoiti-Servitje E . A leading role for the immune system in the pathophysiology of preeclampsia. J Leukoc Biol. (2013) 94:247–57. doi: 10.1189/jlb.1112603, PMID: 23633414

[13] Raguema N Moustadraf S Bertagnolli M . Immune and apoptosis mechanisms regulating placental development and vascularization in preeclampsia. Front Physiol. (2020) 11:98. doi: 10.3389/fphys.2020.00098, PMID: 32116801 PMC7026478

[14] Redman CW Sargent IL . Placental debris, oxidative stress and pre-eclampsia. Placenta. (2000) 21:597–602. doi: 10.1053/plac.2000.0560, PMID: 10985960

[15] Redman CW Tannetta DS Dragovic RA Gardiner C Southcombe JH Collett GP . Review: Does size matter? Placental debris and the pathophysiology of pre-eclampsia. Placenta. (2012) 33:S48–54. doi: 10.1016/j.placenta.2011.12.006, PMID: 22217911

[16] Tannetta D Masliukaite I Vatish M Redman C Sargent I . Update of syncytiotrophoblast derived extracellular vesicles in normal pregnancy and preeclampsia. J Reprod Immunol. (2017) 119:98–106. doi: 10.1016/j.jri.2016.08.008, PMID: 27613663

[17] Redman CWG Staff AC Roberts JM . Syncytiotrophoblast stress in preeclampsia: the convergence point for multiple pathways. Am J Obstet Gynecol. (2022) 226:S907–S27. doi: 10.1016/j.ajog.2020.09.047, PMID: 33546842

[18] Rao A Shinde U Das DK Balasinor N Madan T . Early prediction of pre-eclampsia using circulating placental exosomes: Newer insights. Indian J Med Res. (2023) 158:385–96. doi: 10.4103/ijmr.ijmr_2143_22, PMID: 37987999 PMC10793818

[19] Rao A Subedi R Kundu I Idicula-Thomas S Shinde U Bansal V . Differential proteomics of circulating extracellular vesicles of placental origin isolated from women with early-onset preeclampsia reveal aberrant innate immune and hemostasis processes. Am J Reprod Immunol. (2024) 91:e13860. doi: 10.1111/aji.13860, PMID: 38804582

[20] Deng L Bremme K Hansson LO Blomback M . Plasma levels of von Willebrand factor and fibronectin as markers of persisting endothelial damage in preeclampsia. Obstet Gynecol. (1994) 84:941–5. doi: 10.1053/plac.2000.0560, PMID: 7970473

[21] Powe CE Levine RJ Karumanchi SA . Preeclampsia, a disease of the maternal endothelium: the role of antiangiogenic factors and implications for later cardiovascular disease. Circulation. (2011) 123:2856–69. doi: 10.1161/CIRCULATIONAHA.109.853127, PMID: 21690502 PMC3148781

[22] Goulopoulou S Davidge ST . Molecular mechanisms of maternal vascular dysfunction in preeclampsia. Trends Mol Med. (2015) 21:88–97. doi: 10.1016/j.molmed.2014.11.009, PMID: 25541377

[23] Johnson U Gustavii B . Complement components in normal pregnancy. Acta Pathol Microbiol Immunol Scand C. (1987) 95:97–9. doi: 10.1111/j.1699-0463.1987.tb00014.x, PMID: 3630717

[24] Burwick RM Java A Regal JF . The role of complement in normal pregnancy and preeclampsia. Front Immunol. (2025) 16:1643896. doi: 10.3389/fimmu.2025.1643896, PMID: 40777009 PMC12328418

[25] Bulla R Agostinis C Bossi F Rizzi L Debeus A Tripodo C . Decidual endothelial cells express surface-bound C1q as a molecular bridge between endovascular trophoblast and decidual endothelium. Mol Immunol. (2008) 45:2629–40. doi: 10.1016/j.molimm.2007.12.025, PMID: 18295334 PMC2632959

[26] Agostinis C Bulla R Tripodo C Gismondi A Stabile H Bossi F . An alternative role of C1q in cell migration and tissue remodeling: contribution to trophoblast invasion and placental development. J Immunol. (2010) 185:4420–9. doi: 10.4049/jimmunol.0903215, PMID: 20810993

[27] Richani K Soto E Romero R Espinoza J Chaiworapongsa T Nien JK . Normal pregnancy is characterized by systemic activation of the complement system. J Matern Fetal Neonatal Med. (2005) 17:239–45. doi: 10.1080/14767050500072722, PMID: 16147832 PMC1421513

[28] Derzsy Z Prohászka Z Rigó J Füst G Molvarec A . Activation of the complement system in normal pregnancy and preeclampsia. Mol Immunol. (2010) 47:1500–6. doi: 10.1016/j.molimm.2010.01.021, PMID: 20181396

[29] Buurma A Cohen D Veraar K Schonkeren D Claas FH Bruijn JA . Preeclampsia is characterized by placental complement dysregulation. Hypertension. (2012) 60:1332–7. doi: 10.1161/HYPERTENSIONAHA.112.194324, PMID: 23006730

[30] Halmos A Rigó J Szijártó J Füst G Prohászka Z Molvarec A . Circulating ficolin-2 and ficolin-3 in normal pregnancy and pre-eclampsia. Clin Exp Immunol. (2012) 169:49–56. doi: 10.1111/j.1365-2249.2012.04590.x, PMID: 22670778 PMC3390473

[31] Kestlerova A Feyereisl J Frisova V Mechurova A Sula K Zima T . Immunological and biochemical markers in preeclampsia. J Reprod Immunol. (2012) 96:90–4. doi: 10.1016/j.jri.2012.10.002, PMID: 23131770

[32] Kim EN Yoon BH Lee JY Hwang D Kim KC Lee J . Placental C4d deposition is a feature of defective placentation: observations in cases of preeclampsia and miscarriage. Virchows Arch. (2015) 466:717–25. doi: 10.1007/s00428-015-1759-y, PMID: 25820373

[33] Agostinis C Stampalija T Tannetta D Loganes C Vecchi Brumatti L De Seta F . Complement component C1q as potential diagnostic but not predictive marker of preeclampsia. Am J Reprod Immunol. (2016) 76:475–81. doi: 10.1111/aji.12586, PMID: 27666323

[34] Regal JF Burwick RM Fleming SD . The complement system and preeclampsia. Curr Hypertens Rep. (2017) 19:87. doi: 10.1007/s11906-017-0784-4, PMID: 29046976 PMC5849056

[35] Sarween N Drayson MT Hodson J Knox EM Plant T Day CJ . Humoral immunity in late-onset Pre-eclampsia and linkage with angiogenic and inflammatory markers. Am J Reprod Immunol. (2018) 80:e13041. doi: 10.1111/aji.13041, PMID: 30168226

[36] Agostinis C Zito G Toffoli M Peterlunger I Simoni L Balduit A . A longitudinal study of C1q and anti-C1q autoantibodies in homologous and heterologous pregnancies for predicting pre-eclampsia. Front Immunol. (2022) 13:1037191. doi: 10.3389/fimmu.2022.1037191, PMID: 36439146 PMC9682096

[37] Dijkstra DJ Lokki AI Gierman LM Borggreven NV van der Keur C Eikmans M . Circulating levels of anti-C1q and anti-factor H autoantibodies and their targets in normal pregnancy and preeclampsia. Front Immunol. (2022) 13:842451. doi: 10.3389/fimmu.2022.842451, PMID: 35432365 PMC9009242

[38] Balduit A Agostinis C Mangogna A Zito G Stampalija T Ricci G . Systematic review of the complement components as potential biomarkers of pre-eclampsia: pitfalls and opportunities. Front Immunol. (2024) 15. doi: 10.3389/fimmu.2024.1419540, PMID: 38983853 PMC11232388

[39] Lynch AM Gibbs RS Murphy JR Giclas PC Salmon JE Holers VM . Early elevations of the complement activation fragment C3a and adverse pregnancy outcomes. Obstet Gynecol. (2011) 117:75–83. doi: 10.1097/AOG.0b013e3181fc3afa, PMID: 21173647 PMC5267353

[40] Belmonte B Mangogna A Gulino A Cancila V Morello G Agostinis C . Distinct roles of classical and lectin pathways of complement in preeclamptic placentae. Front Immunol. (2022) 13:882298. doi: 10.3389/fimmu.2022.882298, PMID: 35711467 PMC9197446

[41] Schwaeble WJ Reid KB . Does properdin crosslink the cellular and the humoral immune response? Immunol Today. (1999) 20:17–21. doi: 10.1016/s0167-5699(98)01376-0, PMID: 10081225

[42] Kemper C Atkinson JP Hourcade DE . Properdin: emerging roles of a pattern-recognition molecule. Annu Rev Immunol. (2010) 28:131–55. doi: 10.1146/annurev-immunol-030409-101250, PMID: 19947883

[43] Kouser L Abdul-Aziz M Nayak A Stover CM Sim RB Kishore U . Properdin and factor h: opposing players on the alternative complement pathway “see-saw. Front Immunol. (2013) 4:93. doi: 10.3389/fimmu.2013.00093, PMID: 23630525 PMC3632793

[44] Kouser L Paudyal B Kaur A Stenbeck G Jones LA Abozaid SM . Human properdin opsonizes nanoparticles and triggers a potent pro-inflammatory response by macrophages without involving complement activation. Front Immunol. (2018) 9:131. doi: 10.3389/fimmu.2018.00131, PMID: 29483907 PMC5816341

[45] Rothfield N Ross HA Minta JO Lepow IH . Glomerular and dermal deposition of properdin in systemic lupus erythematosus. N Engl J Med. (1972) 287:681–5. doi: 10.1056/NEJM197210052871402, PMID: 4115613

[46] Ziegler JB Rosen FS Alper CA Grupe W Lepow IH . Metabolism of properdin in normal subjects and patients with renal disease. J Clin Invest. (1975) 56:761–7. doi: 10.1172/JCI108147, PMID: 1159085 PMC301925

[47] Van Essen MF Ruben JM De Vries APJ Van Kooten C Consortium C . Role of properdin in complement-mediated kidney diseases. Nephrol Dial Transplant. (2019) 34:742–50. doi: 10.1093/ndt/gfy233, PMID: 30053164

[48] Tannetta DS Dragovic RA Gardiner C Redman CW Sargent IL . Characterisation of syncytiotrophoblast vesicles in normal pregnancy and pre-eclampsia: expression of Flt-1 and endoglin. PloS One. (2013) 8:e56754. doi: 10.1371/journal.pone.0056754, PMID: 23437230 PMC3577732

[49] Burgoyne LA . The mechanisms of pyknosis: hypercondensation and death. Exp Cell Res. (1999) 248:214–22. doi: 10.1006/excr.1999.4406, PMID: 10094828

[50] Heazell AE Moll SJ Jones CJ Baker PN Crocker IP . Formation of syncytial knots is increased by hyperoxia, hypoxia and reactive oxygen species. Placenta. (2007) 28:S33–40. doi: 10.1016/j.placenta.2006.10.007, PMID: 17140657

[51] Hou L Liu K Li Y Ma S Ji X Liu L . Necrotic pyknosis is a morphologically and biochemically distinct event from apoptotic pyknosis. J Cell Sci. (2016) 129:3084–90. doi: 10.1242/jcs.184374, PMID: 27358477

[52] Homer RS Mc NE . Natural resistance to infectious diseases during pregnancy: possible relationship to serum properdin concentration. Am J Obstet Gynecol. (1961) 81:29–41. doi: 10.1016/S0002-9378(16)36303-7, PMID: 13715633

[53] Pangburn MK . Analysis of the natural polymeric forms of human properdin and their functions in complement activation. J Immunol. (1989) 142:202–7. doi: 10.4049/jimmunol.142.1.202 2909614

[54] Nolan KF Reid KB . Properdin. Methods Enzymol. (1993) 223:35–46. doi: 10.1016/0076-6879(93)23036-M, PMID: 8271963

[55] Cortes C Ohtola JA Saggu G Ferreira VP . Local release of properdin in the cellular microenvironment: role in pattern recognition and amplification of the alternative pathway of complement. Front Immunol. (2012) 3:412. doi: 10.3389/fimmu.2012.00412, PMID: 23335922 PMC3547370

[56] Stover CM McDonald J Byrne S Lambert DG Thompson JP . Properdin levels in human sepsis. Front Immunol. (2015) 6:24. doi: 10.3389/fimmu.2015.00024, PMID: 25699043 PMC4313716

[57] Perdikoulis MV Kishore U Reid KB . Expression and characterisation of the thrombospondin type I repeats of human properdin. Biochim Biophys Acta. (2001) 1548:265–77. doi: 10.1016/S0167-4838(01)00238-2, PMID: 11513971

[58] Blatt AZ Pathan S Ferreira VP . Properdin: a tightly regulated critical inflammatory modulator. Immunol Rev. (2016) 274:172–90. doi: 10.1111/imr.12466, PMID: 27782331 PMC5096056

[59] Ferreira VP Cortes C Pangburn MK . Native polymeric forms of properdin selectively bind to targets and promote activation of the alternative pathway of complement. Immunobiology. (2010) 215:932–40. doi: 10.1016/j.imbio.2010.02.002, PMID: 20382442 PMC2949450

[60] Wang W Irani RA Zhang Y Ramin SM Blackwell SC Tao L . Autoantibody-mediated complement C3a receptor activation contributes to the pathogenesis of preeclampsia. Hypertension. (2012) 60:712–21. doi: 10.1161/HYPERTENSIONAHA.112.191817, PMID: 22868393 PMC4131740

[61] Blakey H Sun R Xie L Russell R Sarween N Hodson J . Pre-eclampsia is associated with complement pathway activation in the maternal and fetal circulation, and placental tissue. Pregnancy Hypertens. (2023) 32:43–9. doi: 10.1016/j.preghy.2023.04.001, PMID: 37088032

[62] Agostinis C Bossi F Masat E Radillo O Tonon M De Seta F . MBL interferes with endovascular trophoblast invasion in pre-eclampsia. Clin Dev Immunol. (2012) 2012:484321. doi: 10.1155/2012/484321, PMID: 22203857 PMC3235499

[63] Yasmin H Agostinis C Toffoli M Roy T Pegoraro S Balduit A . Protective role of complement factor H against the development of preeclampsia. Front Immunol. (2024) 15:1351898. doi: 10.3389/fimmu.2024.1351898, PMID: 38464530 PMC10920295

[64] Chen JY Cortes C Ferreira VP . Properdin: A multifaceted molecule involved in inflammation and diseases. Mol Immunol. (2018) 102:58–72. doi: 10.1016/j.molimm.2018.05.018, PMID: 29954621 PMC7375857

[65] Fearon DT Austen KF . Properdin: binding to C3b and stabilization of the C3b-dependent C3 convertase. J Exp Med. (1975) 142:856–63. doi: 10.1084/jem.142.4.856, PMID: 1185108 PMC2189935

[66] Sharp AN Heazell AE Crocker IP Mor G . Placental apoptosis in health and disease. Am J Reprod Immunol. (2010) 64:159–69. doi: 10.1111/j.1600-0897.2010.00837.x, PMID: 20367628 PMC3025811

[67] Allaire AD Ballenger KA Wells SR McMahon MJ Lessey BA . Placental apoptosis in preeclampsia. Obstet Gynecol. (2000) 96:271–6. doi: 10.1016/s0029-7844(00)00895-4, PMID: 10908776

[68] Leung DN Smith SC To KF Sahota DS Baker PN . Increased placental apoptosis in pregnancies complicated by preeclampsia. Am J Obstet Gynecol. (2001) 184:1249–50. doi: 10.1067/mob.2001.112906, PMID: 11349196

[69] Heazell AE Buttle HR Baker PN Crocker IP . Altered expression of regulators of caspase activity within trophoblast of normal pregnancies and pregnancies complicated by preeclampsia. Reprod Sci. (2008) 15:1034–43. doi: 10.1177/1933719108322438, PMID: 19088373

[70] Ishihara N Matsuo H Murakoshi H Laoag-Fernandez JB Samoto T Maruo T . Increased apoptosis in the syncytiotrophoblast in human term placentas complicated by either preeclampsia or intrauterine growth retardation. Am J Obstet Gynecol. (2002) 186:158–66. doi: 10.1067/mob.2002.119176, PMID: 11810103

[71] Levy R Smith SD Yusuf K Huettner PC Kraus FT Sadovsky Y . Trophoblast apoptosis from pregnancies complicated by fetal growth restriction is associated with enhanced p53 expression. Am J Obstet Gynecol. (2002) 186:1056–61. doi: 10.1067/mob.2002.122250, PMID: 12015537

[72] Endo H Okamoto A Yamada K Nikaido T Tanaka T . Frequent apoptosis in placental villi from pregnancies complicated with intrauterine growth restriction and without maternal symptoms. Int J Mol Med. (2005) 16:79–84. doi: 10.3892/ijmm.16.1.79, PMID: 15942681

[73] Cali U Cavkaytar S Sirvan L Danisman N . Placental apoptosis in preeclampsia, intrauterine growth retardation, and HELLP syndrome: an immunohistochemical study with caspase-3 and bcl-2. Clin Exp Obstet Gynecol. (2013) 40:45–8., PMID: 23724505

[74] Xu W Berger SP Trouw LA De Boer HC Schlagwein N Mutsaers C . Properdin binds to late apoptotic and necrotic cells independently of C3b and regulates alternative pathway complement activation. J Immunol. (2008) 180:7613–21. doi: 10.4049/jimmunol.180.11.7613, PMID: 18490764

[75] Hoffman MC Rumer KK Kramer A Lynch AM Winn VD . Maternal and fetal alternative complement pathway activation in early severe preeclampsia. Am J Reprod Immunol. (2014) 71:55–60. doi: 10.1111/aji.12162, PMID: 24128411 PMC4067768

[76] Gupta A Hasler P Gebhardt S Holzgreve W Hahn S . Occurrence of neutrophil extracellular DNA traps (NETs) in pre-eclampsia: a link with elevated levels of cell-free DNA? Ann N Y Acad Sci. (2006) 1075:118–22. doi: 10.1196/annals.1368.015, PMID: 17108200

[77] O’Flynn J Dixon KO Faber Krol MC Daha MR Van Kooten C . Myeloperoxidase directs properdin-mediated complement activation. J Innate Immun. (2014) 6:417–25. doi: 10.1159/000356980, PMID: 24355864 PMC6741500

[78] Wirthmueller U Dewald B Thelen M Schafer MK Stover C Whaley K . Properdin, a positive regulator of complement activation, is released from secondary granules of stimulated peripheral blood neutrophils. J Immunol. (1997) 158:4444–51. doi: 10.4049/jimmunol.158.9.4444, PMID: 9127010

[79] Staff AC . The two-stage placental model of preeclampsia: An update. J Reprod Immunol. (2019) 134-135:1–10. doi: 10.1016/j.jri.2019.07.004, PMID: 31301487

[80] Knight M Redman CW Linton EA Sargent IL . Shedding of syncytiotrophoblast microvilli into the maternal circulation in pre-eclamptic pregnancies. Br J Obstet Gynaecol. (1998) 105:632–40. doi: 10.1111/j.1471-0528.1998.tb10178.x, PMID: 9647154

[81] Kumar V Stewart JHT . The complement system in human pregnancy and preeclampsia. Front Immunol. (2025) 16:1617140. doi: 10.3389/fimmu.2025.1617140, PMID: 40904461 PMC12401976

