# Supplementary figures and images for: Expression of Properdin, the positive regulator of the Complement Alternative Pathway, at the fetal-maternal interface in Preeclampsia
Source: Front Immunol. 2026 Feb 4;16:1739327. doi: 10.3389/fimmu.2025.1739327 (PMC12913580; doi:10.3389/fimmu.2025.1739327)

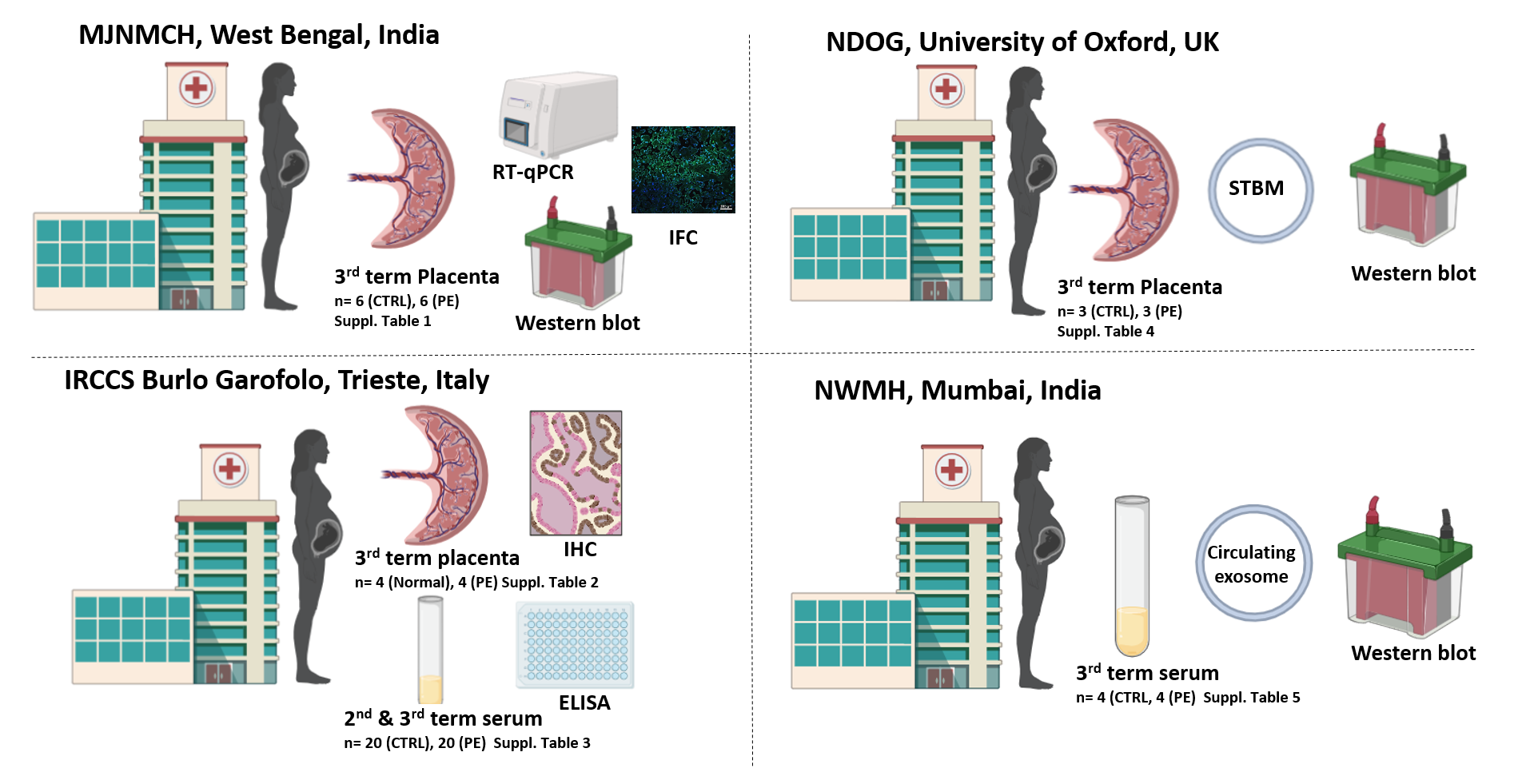

Supplement: SUPPLEMENTARY FIGURE 1 — Graphical description of the multiple cohorts of pregnant women from which serum and placental samples were collected. [file Image1.tif]

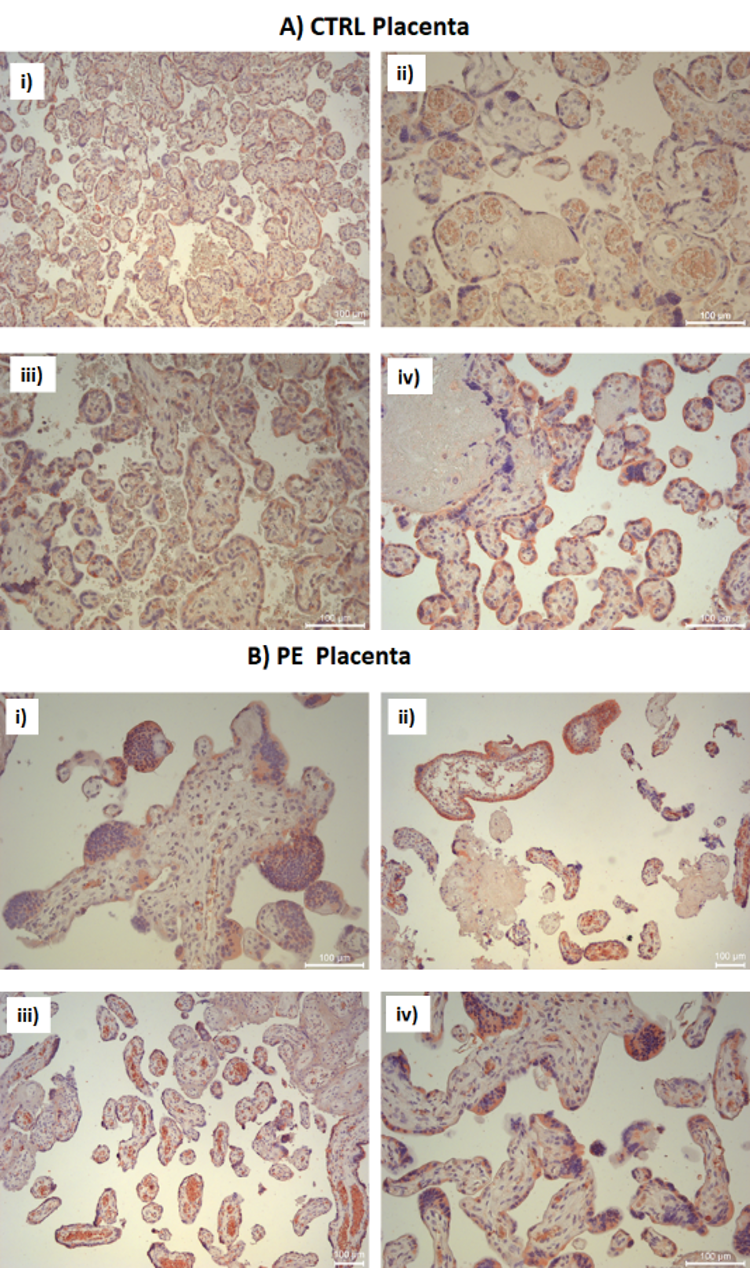

Supplement: SUPPLEMENTARY FIGURE 2 — IHC analysis of properdin in placental tissues from (A) normal (CTRL) and (B) preeclamptic (PE) tissues. Both CTRL and PE placental tissues showed properdin staining, being prominently high in the syncytial knots of PE placentae. [file Image2.tif]
