# Supplementary material for: Expression of Properdin, the positive regulator of the Complement Alternative Pathway, at the fetal-maternal interface in Preeclampsia
Source: Front Immunol. 2026 Feb 4;16:1739327. doi: 10.3389/fimmu.2025.1739327 (PMC12913580; doi:10.3389/fimmu.2025.1739327)
Supplement: Supplementary file 3 [file Table1.docx]

**Supplementary Table 1. Clinical characteristics of the cohort from Maharaja Jitendra Narayan Medical College and Hospital.** PE placentae were from mothers without labour.

Abbreviations: wks, weeks; y, years.

| MJNMCH | **HEALTHY PREGNANCY (CTRL)**  **(n=6)** | **PREECLAMPSIA (PE)**  **(n=6)** |
| --- | --- | --- |
| Age (y) | 25 ± 1.3 | 25 ± 1.6 |
| Gestational age at delivery (wks) | ≥ 36 | ≥ 36 |
| Mode of delivery: |  |  |
| *- Vaginal delivery (%)* | - | - |
| *- Cesarean section (%)* | 100% | 100% |

**Supplementary Table 2. Clinical characteristics of the cohort from IRCCS Burlo Garofolo.** PE placentae were from mothers without labour.

| IRCCS (IHC) | **HEALTHY PREGNANCY (CTRL)**  **(n= 4)** | **PREECLAMPSIA (PE)**  **(n=4)** |
| --- | --- | --- |
| Age (y) | 36.3 ± 2.3 | 28.7 ± 7.8 |
| Gestational age at delivery (wks) | 39.0 ± 2.0 | 30.3 ± 5.4 |
| Mode of delivery: |  |  |
| *- Vaginal delivery (%)* | 66.7% | - |
| *- Cesarean section (%)* | 33.3% | 100% |

Abbreviations: wks, weeks; y, years.

**Supplementary Table 3. Clinical characteristics of the cohort from IRCCS Burlo Garofolo.** PE placentae from caesarean sections are without labour.

| IRCCS (Serum) | **HEALTHY PREGNANCY (CTRL)**  **(n=20)** | **PREECLAMPSIA (PE)**  **(n=20)** |
| --- | --- | --- |
| Age (y) | *n.a.* | *n.a.* |
| Gestational age at diagnosis (wks) | 33.0 | 33.4 |
| Mode of delivery: |  |  |
| *- Vaginal delivery (%)* | 100% | 20% |
| *- Cesarean section (%)* | - | 80% |

Abbreviations: n.a., not available; wks, weeks; y, years.

**Supplementary Table 4. Clinical characteristics of the cohort from Nuffield Department of Obstetrics and Gynaecology, John Radcliffe Hospital, University of Oxford.**

| NDOG | **HEALTHY PREGNANCY (CTRL)**  **(n=4)** | **PREECLAMPSIA (PE)**  **(n=6)** |
| --- | --- | --- |
| Age (y) | 33 ± 1.8 | 35 ± 2.4 |
| Gestational age at delivery (wks) | 39.0 | 37.1 ± 1.3 |
| Mode of delivery: | *n.a.* | *n.a.* |
| *- Vaginal delivery (%)* |  |  |
| *- Cesarean section (%)* |  |  |

Abbreviations: n.a., not available; wks, weeks; y, years.

**Supplementary Table 5. Clinical characteristics of the cohort from Nowrosjee Wadia Maternity Hospital (NWMH), Mumbai**

| NWMH | **HEALTHY PREGNANCY (CTRL)**  **(n=4)** | **PREECLAMPSIA (PE)**  **(n=4)** |
| --- | --- | --- |
| Age (y) | *n.a.* | *n.a.* |
| Gestational age at sampling (wks) | 30.5 ± 2.6 | 30.4 ± 1.9 |
| Mode of delivery: | *n.a.* | *n.a.* |
| *- Vaginal delivery (%)* |  |  |
| *- Cesarean section (%)* |  |  |

Abbreviations: n.a., not available; wks, weeks; y, years.

| **Target** | **Forward primer** | **Reverse Primer** | **Annealing Temp (^o^C)** |
| --- | --- | --- | --- |
| **GAPDH** | 5’- GAT CAT CAG CAA TGC CTC CT-3’ | 5’- GT GGT CAT GAG TCC TTC CA -3’ | 52.0 |
| **Properdin** | 5’- GAATGGGCAGTGCTCTGGAAAG-3’ | 5’- TTGGAGCAGGTGACAGAGCAAG -3’ | 57.2 |
| **C3** | 5’- CCTGCTACTAACCCACCTCC-3’ | 5’- AACAGTGACTGGAACATCCCC-3’ | 54.5 |
| **C5** | 5’- ATGGGCCTTTTGGGAATACTTTG-3’ | 5’- ACATGGCCTGAGGAGTAACTAA-3’ | 54.0 |
| **TNF-α** | 5’-AGCCCATGTTGTAGCAAACC-3’ | 5’-TGAGGTACAGGCCCTCTGAT-3’ | 54.2 |
| **TGF-β** | 5’-GTACCTGAACCCGTGTTGCT-3’ | 5’-GTATCGCCAGGAATTGTTGC-3’ | 53.88 |
| **VEGF** | 5’-TGCAGATTATGCGGATCAAACC-3’ | 5’-TGCATTCACATTTGTTGTGCTGTAG-3’ | 55.2 |

**Supplementary Table 6. Details of gene primers.**
